# Spectrophotometric Determination of the Thermodynamic *p*K Value of Picric Acid in Water at 25 °C

**DOI:** 10.6028/jres.067A.024

**Published:** 1963-06-01

**Authors:** Marion Maclean Davis, Maya Paabo

## Abstract

The thermodynamic *p*K value of picric acid was determined spectrophotometrically in water containing hydrochloric acid to repress the ionization. The *p*K value 0.33 (*K* ≈ 0.46) was obtained from data at 450 m*μ*. Attempts to determine the *p*K value by potentiometric titrations of picric acid and by spectrophotometric measurements of picric acid solutions in the near-saturation range did not yield satisfactory results. The new *p*K value is compared with previously published values.

## 1. Introduction

Because of the wide-ranging importance of picric acid, numerous attempts have been made to determine its ionization constant in water. Table 1 summarizes ionization constants or *p*K values which have been reported in the literature, obtained by catalytic, conductance, distribution, or spectrophotometric methods.^1^ The values obtained for *K* range from about 0.15 to 0.6, the corresponding *p*K range being about 0.8 to 0.2.

The principle of additivity of substituent effects, which has been applied successfully in calculating approximate *p*K values for some of the *meta*- and *para*-substituted benzoic acids (for example, see [1, 2]),^2^ cannot be used for a dependable estimate of the *p*K values of di- and trinitrophenols, because the calculated acidic strengths are less than those determined experimentally—in some cases, very much less [3 to 5]. For picric acid, several different estimated *p*K values are obtained on using different combinations of the following experimentally obtained *p*K values: Phenol (10.00), *m*-cresol (10.09), 3.5-xylenol (10.19), *o*-nitrophenol (7.21), *p*-nitrophenol (7.15), 2,4-dinitrophenol (4.10), 2,6-dinitrophenol (3.71), 2,4,6-trinitro-*m*-cresol (0.81), and 3.5-dimethylpicric acid (1.38). The variability of the calculated *p*K values is evident from the following examples:

(1) The *p*K value 1.57 is obtained by using *p*K values for phenol, *o*-nitrophenol and *p*-nitrophenol.
(2) By subtracting the numerical difference between the *p*K values for phenol and *o*-nitrophenol from the *p*K value for 2,4-dinitrophenol one obtains 1.31.
(3) Subtracting the numerical difference between the *p*K values for phenol and *p*-nitrophenol from the *p*K value for 2,6-dinitropnenol gives the value 0.86.
(4) Subtracting 0.09 from the experimental *p*K value for trinitro-*m*-cresol gives 0.72.
(5) Subtraction of 0.19 from the experimental value found for dimethylpicric acid gives 1.19.

Bearing in mind that the experimental *p*K values for the dinitrophenols are all lower than the values calculated assuming additivity of substituent effects, one may reasonably expect the experimental *p*K for picric acid, also, to be lower than values calculated in (1) to (3). The *p*K values calculated in (4) and (5) likewise set upper limits for the *p*K value to be expected for picric acid. Dimethylpicric acid, in particular, undoubtedly is weakened by steric inhibition of resonance of nitro groups with the ring, caused by adjacent methyl groups—this follows from well-known theoretical considerations, as well as from its absorption spectrum [4]. It is very likely that trinitro-*m*-cresol, too, is weakened by steric inhibition of resonance, though not to the same degree as dimethylpicric acid.

The two following additional modes of calculating a *p*K value for picric acid,^3^ which use only experimental *p*K values for nitrophenols, should give a better estimate of the range within which the true *p*K value falls:

(6) Subtracting the difference between the *p*K value for *o*-nitrophenol and that for 2,4-dinitrophenol from the value for 2,6-dinitrophenol gives 0.60.^4^
(7) Subtracting the difference between the *p*K values for dimethylpicric acid and trinitro-*m*-cresol from the value for trinitro-*m*-cresol gives 0.24.

This paper reports the results obtained in applying to picric acid the spectrophotometric procedure used in determining the *p*K values of 3,5-dimethylpicric acid [4] and 2,4,6-trinitro-*m*-cresol [5], in which hydrochloric acid is added to repress the ionization of dilute aqueous picric acid. Absorbance measurements were also made for aqueous picric acid at higher concentrations. Attempts to measure the *p*K by potentiometric titration are discussed briefly.

## 2. Experimental Procedures

### 2.1. Materials

A commercial high grade of *picric acid* was recrystallized several times from benzene-cyclohexane or water, the final time from water, forming long needles. Preliminary drying to constant weight at room temperature was accomplished by leaving the crystals for approximately four hours in a desiccator through which dried air passed continually. No further measurable loss in weight occurred on heating for an hour at 100 to 110 °C. The melting point was 121.5 to 122.0 °C. The values for percent purity obtained by potentiometric weight-titrations of two samples were 99.97 and 100.02.

The *hydrochloric acid* and *sodium hydroxide* were commercial materials of highest grade, stated to meet A.C.S. specifications.

### 2.2. Attempt to Determine *p*K by Potentiometric Titrations

Solutions of picric acid about 0.004 *M* or 0.044 *M* were titrated with approximately 0.46-*M* sodium hydroxide, using glass and sleeve-type calomel electrodes. The method of calculation was the same as in the titrimetric determination of the *p*K of 2,6-dichlorobenzoic acid [2]. Before the titrations the electrode system was checked with NBS 0.05-*M* potassium acid phthalate buffer (*p*H 4.01) and 0.05-*M* potassium tetroxalate buffer (*p*H 1.65). The initial *p*H values ranged from 1.50 to 1.54, and the average *p*K values from three experiments were 0.78, 0.86, and 0.74. These are *not* considered reliable values. In the first place, *p*K values calculated by this method are highly sensitive to the magnitude of the experimental *p*H values, and these were not reproducible enough. There was evidence of a tendency for picrate to crystallize near the sleeve of the calomel electrode during titrations. A second difficulty concerns the standardization of the instrument with buffer solutions before the titrations. The *p*H range near 1.50, which is vital for this method of determining the *p*K of picric acid, is outside the range of *p*H values in which the Hitchcock and Taylor scale of *p*H values (obtained with buffer solutions using cells with liquid junction) and the NBS scale (obtained using cells without liquid junction) are in excellent agreement [6]. Near *p*H 1.50, the two scales differ by 0.04 *p*H unit.

### 2.3. Determination of the *p*K Value by Spectrophotometry

Absorbances were obtained with a Beckman Model DU quartz spectrophotometer, equipped with a thermostated cell compartment [7]. The absorption cells were the same accurately made, demountable cells of various lengths used in other spectrophotometric studies [8]. When solutions contained hydrochloric acid or sodium hydroxide, the same concentration of acid or alkali was present in the sample cell and the reference cell. To increase the precision of wavelength settings, the spectrophotometer is provided with an auxiliary indicator line, as recommended in [9]. The absorbance values used are the average of at least five independent settings, which were highly reproducible, even on a steep slope of an absorption curve.

#### a. Determination of *p*K in Hydrochloric Acid Solutions of Picric Acid

In picric acid solutions as dilute as 5×10^−5^ *M* the concentration of nonionized molecules is negligibly small unless a stronger acid is added to repress the ionization. The *p*K value was calculated from the equation

$$\begin{array}{l} pK=-\log\;[H^{+}]-2\;\log\;\gamma_{\pm }\\ \;-\log\;[(D-D_{1})/(D_{2}-D)]. \end{array}$$ (1)

In this equation [H^+^] denotes the total hydrogen ion concentration in moles per liter, and *γ_±_* is taken as the mean activity coefficient of aqueous hydrochloric acid of equivalent molarity (see [4], footnote 4). The symbols *D*_1_, *D*_2_, and *D* apply to any picric acid solution of a given stoichiometric concentration, *D*_1_ being the spectral absorbance (optical density) when the acid is present entirely as HPi, *D*_2_, the absorbance when it is present entirely as Pi^−^, and *D*, the absorbance when conversion of HPi to Pi^−^ or vice versa is only partial. The use of eq (1) involves the usual convention that *γ*_+_·*γ*_−_ = *γ*_±_^2^ for hydrochloric acid, as well as the assumptions that *γ*_HPi_=1 and *γ*_Pi_**_−_** is the same as *γ***_±_** for hydrochloric acid.

A recognized problem in determining spectrophotometrically the values of acids as strong as picric acid is the difficulty in determining *D*_1_ values. In the high concentrations of mineral acid needed for repressing the ionization, additional effects on the spectral absorption seem prone to occur. In the hope of meeting this difficulty, measurements were made not only at 355 m*μ*, which is near the wavelength of maximum absorption of Pi^−^, and at 400 m*μ*, which is on a shoulder of the absorption curve for Pi^−^, but also at 450 m*μ*, where it seemed fairly certain that *D*_1_ would equal zero.

#### b. Attempt to Determine *p*K Using More Concentrated Aqueous Solutions

Using the shortest available absorption cells (0.01-cm), absorbance measurements were made at 450 m*μ* for five picric acid solutions ranging from 0.025 *M* to 0.050 *M*, with and without additions of sodium hydroxide. The absorbance values indicated that in all of these solutions more than 90 percent of the solute was present as Pi^−^. An attempt was made to calculate *p*K by eq (1) (with the same assumptions as before). These calculations, which indicated that *p*K may be about 0.4 or less, cannot be considered reliable, both because of experimental uncertainties and because of the uncertainty as to how much the assumed activity coefficients differ from the true values.

## 3. Results and Discussion

### 3.1. Spectral Absorption Curves

**Figure 1:**
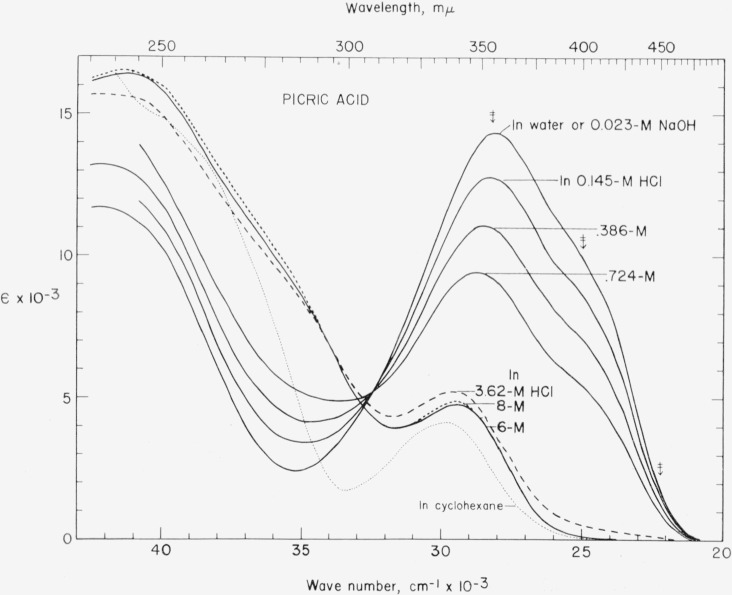
Spectral absorption curves of picric acid (5×10^−5^ *M*) in water, in aqueous alkali (approx. 0.023-M, p*H*>12), and in aqueous acid (*HCl*, ranging from 0.145 M to 8 M). t=25 °C. The faintly dotted line indicates the spectral absorption of picric acid in cyclohexane [10]. The arrows indicate the wavelengths (355 m*μ*, 400 m*μ*, and 450 m*μ*) used in determining *p*K. The absorption in water and 0.023-M NaOH agreed very closely in the entire spectral range measured.

Figure 1 shows a series of absorption curves obtained with 5×10^−5^-*M* picric acid in water, in 0.023-*M* sodium hydroxide, and in aqueous hydrochloric acid ranging from 0.145 *M* to 8 *M.* The faintly dotted line shows the spectral absorption of picric acid in cyclohexane [10]. The arrows indicate the three wavelengths used in determining *p*K.

It is generally accepted that ionization of picric acid is essentially complete in solutions as dilute as 5×10^−5^ *M.* This belief finds support in our experiments, in which the absorption curves obtained in water and in 0.023-*M* sodium hydroxide (*p*H>12) agreed very closely. It was thought desirable to limit the excess of alkali, as a large concentration causes an irreversible deepening of the color.^5^ The alkali was added slowly, with stirring, to avoid momentary large excesses.

For all the aqueous solutions containing 0.724-*M* hydrochloric acid or less, there is a well-marked isosbestic point at approximately 307 m*μ*, *ϵ* = 5050. The curves for the three highest concentrations of hydrochloric acid do not pass through this isosbestic point, but appear to be shifted toward the ultraviolet. The curves obtained with 6-*M* and 8-*M* hydrochloric acid are very similar at wavelengths longer than about 280 m*μ*, but in the region from about 320 m*μ* to 350 m*μ* there is a suggestion of the kind of behavior observed in determining the *p*K of dimethylpicric acid [4], when progressive decreases in the absorption on the first additions of hydrochloric acid were followed by increases in the absorption at high concentrations of hydrochloric acid.

As is well known, in the near ultraviolet and visible regions the absorption curve for aqueous picrate appears to be the envelope of at least two overlapping bands, one near 355 m*μ* and the other in the vicinity of 400 m*μ*.^6^ In some solvents, these bands are more clearly distinguishable and, moreover, their positions vary with the nature of the cation (for example, [14, 15]). The absorption of the nonionized acid is also affected by the nature of the medium (see fig. 1 and [14, 15]). Physical constants determined from absorbance measurements in a region of overlapping bands could be in error if, for example, one component band were a “blue-shift” band and another, a “red-shift” band, or if both were “red-shift” bands not equally affected by a changing medium.^7^ In recent years attempts have been made to account theoretically for strong absorption bands of nitroaromatic compounds, including mono- and dinitrophenols and picric acid. For example, it has been suggested that the absorption band of picrate ion near 353 m*μ* and the band showing as a shoulder near 400 m*μ* are intramolecular charge-transfer bands, in which the phenyl and nitro groups act as electron-donor and electron-acceptor groups, respectively (see [19] and references cited). This is recognized as being only a partial interpretation, which does not take into account such factors as inter- and intramolecular hydrogen bonding and steric inhibition of resonance of substituent groups with the benzene ring.

### 3.2. Results of *p*K Determinations in Aqueous Hydrochloric Acid

#### a. *p*K Value Determined at 450 m*μ*

Table 2 summarizes the results obtained by calculating *p*K from absorbance data at 450 m*μ*. The equation used and the assumptions made about activity coefficients were discussed in section 2.3a. At 450 m*μ* the additional assumption was made that *D*_1_*=0*, justification for which is as follows:

1. The absorbance of picric acid in 6-*M* and 8-*M* hydrochloric acid is negligibly small at 450 m*μ* and even shorter wavelengths (fig. 1).
2. Under comparable experimental conditions picric acid dissolved in cyclohexane (see fig. 1) or benzene [15] has negligibly small absorbance in this region.
3. Nonionized 2,4-dinitrophenol does not absorb measurably at wavelengths as long as 450 m*μ*; the close similarity of the absorption curves of 2,4-dinitrophenol and picric acid in both acidic and alkaline solutions has been pointed out [11].

Averaging all of the results from three independent experiments gives 0.33 as the *p*K value of picric acid. (The average obtained by including only the *p*K values obtained for C_HCl_=0.241 *M* or greater, where the values of log [*D*/*(D_2_−D*)] are in the best range, is not significantly different from the average based on all 26 values.)

The good agreement of the results was unexpected, considering the uncertain validity of assumptions made in applying eq (1) and the fact that the wavelength of measurement was on a steep slope instead of on or close to the head of an absorption band (which is generally considered desirable, when feasible). However, as noted in section 2.3, no difficulty was experienced in obtaining absorbance values of high precision.

As table 2 shows, the stoichiometric concentration of picric acid was varied 100-fold in a dilute range, and the concentration of hydrochloric acid extended from about 0.05-*M* to 0.97-*M*. Since the *p*K values obtained under these conditions show no perceptible trend, it seems justifiable to conclude that the *p*K value adopted is valid at zero ionic strength.

#### b. *p*K Values Obtained From Data at 355 m*μ* and 400 m*μ*

The spectral shifts in the most highly concentrated hydrochloric acid solutions (see sec. 3.1) interfere with experimental determination of *D*_1_ values at 355 m*μ* and 400 m*μ*. A general idea of their probable magnitudes can be obtained from the appearance of the curves and the comparative absorbance values at all three wavelengths. *p*K values were calculated assuming two different values of *D*_1_ which were thought to cover the most likely range of values. The results are presented in table 3.

While the values of *p*K obtained from the absorbance data at 355 m*μ* and 400 m*μ* are in the vicinity of 0.3, in agreement with the results obtained at 450 m*μ*, the values in table 3 show a gradual increase in magnitude with increasing concentration of the hydrochloric acid. Conceivably this behavior occurred for one or more of the following reasons:

1. Adoption of values for *D*_2_ that were a little too low. The values adopted were believed to be close to the correct values, but, as table 3 shows, at 355 m*μ* an increase of only 0.005 in the value of *D*_2_ can bring the *p*K values into fairly good agreement with the series obtained at 450 m*μ* (see values enclosed in brackets).
2. Overlapping absorption bands of picrate ion that are not equally affected by the progressive changes in the medium (see sec. 3.1).
3. The gradual hypsochromic (“blue”) shift in the absorption curve of nonionized picric acid.
4. Slight changes in the spectral absorption resulting from interaction of picric acid with trace impurities in the hydrochloric acid.

### 3.3. Concluding Discussion

As indicated above, attempts to determine the *p*K of picric acid in water by potentiometric titration (sec. 2.2) or by applying spectrophotometry to nearly saturated solutions (sec. 2.3b) did not yield results that were considered reliable.

However, the consistent spectrophotometric results obtained at 450 m*μ* with picric acid solutions covering the range 5×10^−5^ *M* to 5×10^−3^ *M*, in the presence of approximately 0.05-*M* to 1-*M* hydrochloric acid, point to the *p*K value 0.33 at 25 °C. The results obtained at 355 m*μ* and 400 m*μ*, though less consistent, support this choice. The value 0.33 is fairly close to three spectrophotometric values obtained earlier at 20° (table 1, footnote h) and at 25° (table 1, footnotes j and 1).

It would be gratifying if this value agreed closely with the latest reported value (*p*K approx. 0.71), obtained from accurate conductance measurements (table 1, footnote k). One may speculate as to whether spectrophotometry and conductometry will always yield the same *p*K value, in view of recent reports ([20, 21] and references cited) that flash photolysis of certain derivatives of phenol produces shortlived excited states of considerably enhanced acidity. However, one of the *p*K values obtained by conductance (table 1, footnote i) is not far from the spectrophotometric value obtained in this work, and, as shown in table 1, *p*K values calculated from conductance measurements depend on assumptions made (for example, table 1, footnote d). Moreover, the latest conductance measurements covered a rather restricted range of concentrations of picric acid (2.358×10^−3^ *M* to 4.410×10)^−3^ *M*).

## Figures and Tables

**Table 1:** Ionization constants previously reported for picric acid

Temp. °C	Range of conens.^a^	Method of measurement^b^	*K*^c^	Equiv. value of *p*K
				
18	0.002079 to 0.03342	Dist.	d0.164	0.785
25	........	Cond.	e~.2	...........
25	0.03 to 0.05	Cat.	.32	f.506
25	.00997 to .0399	Spec.	^*^^g^.151	^*^.820
20	.00194 to .00204	Spec.	b.381	.419
25	.000187 to .004347	Cond.	i.60±35%	.22 (0.41 to 0.09)
25	Approx. .00005	Spec.	^*^.51	^*^^j^0.29
25	.002358 to .004410	Cond.	^*^^k^.196	^*^.708
25	................	Spec.	.42	l.38

a All concentrations appear to have been in molar units.

b Abbreviations used: “Cat.,” catalytic effect on the hydrolysis of ethyl acetate as compared with that of HCl; “Cond.,” conductance; “Dist.,” distribution between water and benzene; “Spec.,” spectrophotometric.

c An asterisk signifies that *K* and *p*K are claimed to be *thermodynamic* values.

d V. Rothmund and C. Drucker, Z. physik. Chem. **46**, 827 (1903). Dippy, Hughes, and Laxton (see footnote k), who evidently recalculated *K*, cite this value as 0.23, which is equivalent to *p*K 0.64. E. Schreiner (see footnote f) obtained *p*K=0.526 at 25° from Rothmund and Drucker’s data.

e S. M. Neale, Trans. Faraday Soc. **17**, 505 (1921). He used λ_0_=376, and observed a trend from *K*=0.299 for *v*=32 liters to 0.155 at *v*=1280 liters.

f E. Schreiner, Z. anorg. allgem. Chem. **138**, 311 (1924). Assumptions made: HCl is completely dissociated; H^+^ is associated with 10 H_2_O; Pi^−^ and HPi are unhydrated. In three expts. with C_HPi_=0.05, 0.04, 0.03, he obtained for −log *K*_0_ the respective values 0.466, 0.534, 0.517. Emf measurements at 18° yielded *p*K=0.540.

g H. von Halban and L. Ebert, Z. physik. Chem. **112**, 359 (1924). From measurements at 450 m*μ* (?). HPi solutions of three concentrations were used, as well as data in 0.116-*N*HCl, and the results were extrapolated by the method of least squares.

h H. von Halban and M. Seiler, Helv. chim. acta **21**, 385 (1938). From measurements at 436 m*μ*. Fourteen measurements were made with C_HPi_ ranging from 0.00194 to 0.00204, C_HCl_ ranging from 0.0084 to 0.0117. When their data are treated as in this paper, the average *p*K value obtained is 0.355.

i G. Kortüm and H. Wilski, Z. physik. Chem. (n.f.) **2**, 256 (1954); prelim. note, G. Kortüm, Z. Elektrochem. **57**, 874 (1953). They used λ_0_=380.44, consider optical results to be surer.

j R. G. Bates and G. Schwarzenbach, Experientia **10**, 482 (1954). From data at 356 m*μ*. As in this work, hydrochloric acid was added to repress ionization of picric acid, but larger amounts were added (C_HCl_, 0.1 molal to 4 molal). The same equation (see eq (1)) was used in calculating *p*K Because of uncertainty about the value of *D*_1_, *p*K values were calculated with eight assumed values of *D*_1_, then plotted against the molality of hydrochloric acid. The best *D*_1_ value was considered to be the one providing the best linear relation between *p*K and the molality of HCl.

k J. F. J. Dippy, S. R. C. Hughes, and J. W. Laxton, J. Chem. Soc. (London) 1956, 2995. Solutions of five concentrations were measured. They used λ_0_=385.5 (obtained by the extrapolation method of R. M. Fuoss (J. Am. Chem. Soc. 57, 488 (1935)).

l Unpublished result of T. Riley and F. A. Long, cited by A. C. McDougall and F. A. Long, J. Phys. Chem. 66, 429 (1962). Experimental conditions were not given.

**Table 2:** p*K* of picric acid in water at 25 °C from data at 450 mμ^a^

Molar concentration of HCl	*D*	$\log\;\frac{D}{D_{2}-D}$		−log[H^+^]	−2 log y_±_	*p*K

Expt. 1. C_HPi_ = 5.004×10^−3^ *M, b* = 5 cm						
0.0966	0.430	0.870	1.015		0.196	0.341
.145	.410	.721	0.839		.218	.336
.193	.392	.611	.714		.230	.333
.290	.359	.444	.538		.242	.336
.386	.330	.320	.413		.244	.337
.483	.304	.218	.316		.242	.340
.579	.284	.143	.237		.236	.330
.724	.250	.021	.140		.222	.341
.821	.230	−.050	.086		.210	.346
.917	.214	−.107	.038		.195	.340
.966	.206	−.137	.015		.186	.338

Expt. 2. C_HPi_=5.005×10^−5^ *M*, *b* = 5 cm						
0.193	0.392	0.611	0.714		0.230	0.333
.241	.375	.521	.618		.238	.335
.483	.308	.233	.316		.242	.325
.604	.277	.117	.219		.235	.337
.845	.229	−.053	.073		.206	.332

Expt. 3. C_HPi_=5.002×10^−3^ *M, b*=0.05 cm						
0.0483	0.452	1.100	1.276		0.164	0.340
.0966	.428	0.853	0.996		.198	.341
.107	.425	.829	.955		.202	.328
.171	.401	.664	.757		.226	.319
.241	.374	.516	.611		.238	.333
.321	.352	.413	.488		.243	.318
.385	.332	.328	.411		.244	.327
.483	.307	.230	.313		.242	.325
.579	.284	.143	.235		.236	.328
.676	.261	.061	.168		.227	.334

Average *p*K						0.334
*K*						0.46
Relative standard deviation, *%*						2.1

a The ionic dissociation constant was derived using the equation
$pK=-\log\;[H^{+}]-2\;\log\;y_{\pm }-\log\;[(D-D_{1})/(D_{2}-D)].$ (1) See the text for discussion of assumptions made about activity coefficients. The symbols *D*_1_, *D*_2_, and *D* denote the spectral absorbances (optical densities) of solutions containing the same stoichiometric concentration of picric acid present as nonionized molecules, ionized molecules, or mixtures of the two, respectively. At 450 m*μ*, *D*_2_ was found to have the value 0.488 and *D*_1_ was assumed to be zero. The symbol *b* denotes the optical cell length.

**Table 3:** *p*K of picric acid in water at 25 °C from data at 355 m*μ* and 400 m*μ*^a^

Molar concentration of HCl	λ=355 m*μ*; *D*_2_=0.720			λ=400 m*μ*; *D*_2_=0.498		
	*D*	Calcd. *p*K, assuming that		*D*	Calcd. *p*K, assuming that	
		*D*_1_=0.182^b^	*D*_1_=0.211^c^		*D*_1_ =0.003^b^	*D*_1_ =0.045^d^

Expt. 1. C_HPi_ = 5.004×10^−5^ *M; b* = 1 cm						
0.0966	0.664	0.28 [0.31]	0.30	0.451	0.23	0.28
.145	.641	.29 [.32]	.32	.430	.26	.30
.193	.622	.29 [.31]	.32	.414	.26	.30
.290	.587	.30 [.31]	.33	.383	.26	.31
.386	.554	.31 [.32]	.34	.354	.27	.32
.483	.526	.31 [.32]	.35	.328	.28	.34
.579	.498	.32 [.33]	.36	.304	.28	.35
.724	.463	.33 [.33]	.37	.271	.29	.36
.821	.444	.32 [.33]	.37	.253	.29	.37
.917	.423	.32 [.33]	.38	.235	.29	.37
.966	.416	.32 [.32]	.37	.227	.28	.37

Expt. 2. C_HPi_ = 5.005×10^−5^ *M; b* = 1 cm						
0.193	0.620	0.30 [.32]	0.33	0.410	0.28	0.33
.241	.601	.31 [.33]	.34	.395	.28	.32
.483	.525	.31 [.32]	.35	.327	.28	.34
.604	.493	.32 [.33]	.36	.299	.28	.35
.845	.439	.32 [.32]	.37	.249	.28	.37

Expt. 3. C_HPi_ = 5.002×10^−3^ *M; b*=0.01 cm						
0.0483	0.688	0.24 [.30]	0.27	0.470	0.22	0.26
.0966	.669	.21 [.25]	.24	.449	.24	.28
.107	.669	.20 [.24]	.23	.447	.22	.26
.171	. 634	.26 [.29]	.29	.421	.25	.30
.241	.607	.27 [.29]	.30	.395	.27	.32
.321	.581	.27 [.29]	.31	.374	.26	.31
.385	.564	.26 [.28]	.30	.358	.25	.30
.483	.531	.29 [.30]	.33	.330	.26	.32
.579	.506	.29 [.30]	.33	.306	.27	.34
.676	.482	.30 [.31]	.34	.286	.27	.34

a Calculated by the same equation as in table 2, using the same values of −log   [H^+^] and −2 log *γ*_±_.

b This is the experimental *D*_1_ value obtained in 6-*M* and 8-*M* HCl. The values in brackets were calculated assuming *D*_2_=0.725.

c This is the *D*_1_ value obtained by shifting the *D*_1_ curve for 8-*M* HCl 5 m*μ* toward longer wavelengths, so that it will pass through the isosbestic point; that is, it is the experimental value for 350 m*μ*.

d This is an arbitrarily selected value.

## Footnotes

1 A few attempts have been made to determine *p*K by emf procedures. The results are not included in table 1, either because they were considered unsatisfactory or because they were not supported by any experimental details or data.

2 Figures in brackets indicate the literature references at the end of this paper.

3 These were suggested by Donald D. Wagman.

4 The same result is obtained, of course, by subtracting the difference between the *p*K values for a-nitrophenol and 2,6-dinitrophenol from the *p*K value for 2,4-dinitrophenol.

5 This reaction is discussed in [11, 12].

6 In cases like this, Vandenbelt and Henrich’s discussion and illustrations of overlapping absorption bands [13] are helpful in visualizing probable locations of the principal component bands.

7 Recent textbooks on absorption spectroscopy, such as [16 to 18], discuss blue- and red-shifts, referring to original papers.
